# Supplementary material for: Complex‐centric proteome profiling by SEC‐SWATH‐MS
Source: Mol Syst Biol. 2019 Jan 14;15(1):e8438. doi: 10.15252/msb.20188438 (PMC6346213; doi:10.15252/msb.20188438)
Supplement: Supplementary file 7 — Dataset EV6 [file MSB-15-e8438-s007.zip › feature_plots_bioplex/Q9Y2T4.pdf]

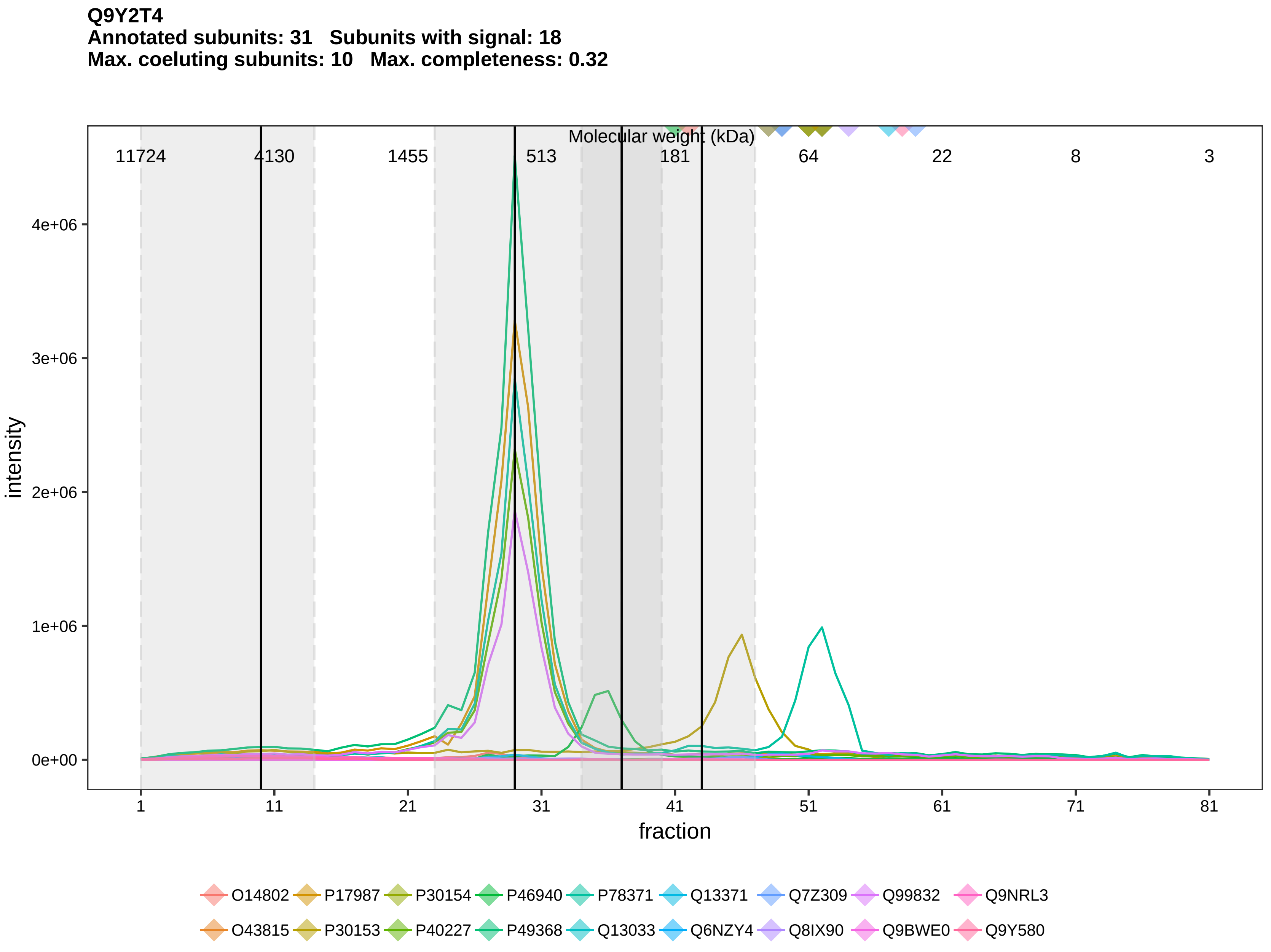
Q9Y2T4
Annotated subunits: 31 Subunits with signal: 18
Max. coeluting subunits: 10 Max. completeness: 0.32
Molecular weight (kDa)
11724
4130
1455
513
181
64
22
8
3
intensity
4e+06
3e+06
2e+06
1e+06
0e+00
fraction
1
11
21
31
41
51
61
71
81
O14802
P17987
P30154
P46940
P78371
Q13371
Q7Z309
Q99832
Q9NRL3
O43815
P30153
P40227
P49368
Q13033
Q6NZY4
Q8IX90
Q9BWE0
Q9Y580
